# An endangered cave-roosting insectivorous bat supports strongly distinct macro-invertebrate communities in caves of an oceanic island

**DOI:** 10.7717/peerj.21622

**Published:** 2026-08-12

**Authors:** Yogishah Bunsy, Christian E. Vincenot, François Benjamin Vincent Florens

**Affiliations:** 1 Tropical Island Biodiversity, Ecology and Conservation Pole of Research, Department of Biosciences and Ocean Studies, Faculty of Science, University of Mauritius, Le Réduit, Mauritius; 2 Complex Systems Group (NEXUS::CSR), Department of Engineering, Faculty of Science, Technology and Medicine, University of Luxembourg, Esch-sur-Alzette, Luxembourg; 3 Island Bat Research Group (IBRG), Kyoto, Japan

**Keywords:** Allochthonous production, Alpha diversity, Bat conservation, Bat guano, Beta diversity, Cave biodiversity, Chiroptera, Lava tube caves, Mauritius, *Mormopterus acetabulosus*

## Abstract

Cave ecosystems are often underrepresented in global conservation management, despite frequently holding diverse biota. As key providers of allochthonous nutrient, cave-roosting bats are important in sustaining cave food webs through guano deposits. However, bat populations globally are declining, thereby threatening cavernicolous biodiversity. Oceanic island caves, in particular, face elevated risks due to their additional isolation which may be expected to increase vulnerability of their associated biodiversity to extinction. Understanding the influence of cave-roosting bat on eventual cave-dependent biodiversity, especially where a single cave-roosting bat species exists, matters for predicting possible cascading effects of loss or gain of cave-roosting bat colonies. We compared macro-invertebrate diversity between lava tube caves with and without the endemic insectivorous bat, *Mormopterus acetabulosus* (Molossidae) across Mauritius, sampling from cave entrance to deep sections and across guano piles. Four caves with permanent bat colonies and four unoccupied caves were surveyed. Macro-invertebrates were collected using pitfall traps and direct collection, and identified to the lowest taxonomic level possible. Species richness, community composition, and beta diversity were compared between bat-occupied and unoccupied caves. Macro-invertebrate richness, abundance, and diversity increased significantly with bat colony size. The three metrics dropped further from guano piles, while light intensity had a positive effect. Community composition differed significantly between bat-occupied caves and unoccupied caves. Allochthonous nutrients from bats substantially boost cave biodiversity, which include possible endemic cave specialists. Bat guano acts as a keystone resource, shaping invertebrate communities, fostering higher species richness and abundance. The drop in diversity away from guano piles reflects nutrient limitation and spatial heterogeneity, while the near absence of invertebrates in unoccupied caves shows that resource availability drives community structure. Our results highlight *M. acetabulosus* as plausible ecosystem engineers that provide guano “islands” whose size and numbers influence cave invertebrate diversity, reflecting island biogeography theory. Conservation measures should prioritise bat-occupied caves, particularly those with large or multiple guano piles. This is important as caves in Mauritius are currently neglected in conservation management strategies. Legal protection of bat roosts, and restricting access to bat colonies and guano piles could be considered. Integrating these measures into conservation policies can prevent biodiversity declines and provides a model for other islands approaching Mauritius’ levels of rapid urbanisation and high population density.

## Introduction

Bats (Chiroptera) tend to be disproportionately represented in island mammal faunas (Fleming & Racey, 2009; Jones et al., 2009; Conenna et al., 2017). Bats constitute one-fifth of the global mammalian diversity, with 1,500 species now recognised (Simmons & Cirranello, 2026), ∼60% of which occur on oceanic islands to which a quarter of all bat species are endemics (Conenna et al., 2017). Bats play key ecological roles like pollination (Fleming, Geiselman & Kress, 2009), seed dispersal (Muscarella & Fleming, 2007), insect pest suppression (Russo, Bosso & Ancillotto, 2018) and nutrient transfer (Duchamp, Sparks & Swihart, 2010), and such roles are magnified on islands owing to their generally low species redundancy (Kunz et al., 2011; McConkey & Drake, 2015; Valido & Olesen, 2023). However, human-induced land-use change (Frick, Kingston & Flanders, 2020), invasive species (Rocha, 2015) and direct persecution (Vincenot, Florens & Kingston, 2017; Kingston, Florens & Vincenot, 2023) amplify extinction risk in island bats, such that around half of island species are threatened (Jones et al., 2009; Conenna et al., 2017; Frick, Kingston & Flanders, 2020).

Cave habitats are often small and far apart, mimicking traits that usually characterise islands (Frick, Kingston & Flanders, 2020; Schrader et al., 2021), particularly for non-volant cave-dependent organisms (Balogh et al., 2020). Some 44% of threatened bats depend on caves for their primary roosting sites (IUCN, 2026). These bat-occupied caves become critical habitats that often support unique biodiversity (Culver & Pipan, 2019). Bat guano usually provides the main allochthonous input of carbon into caves, sustaining the trophic network of invertebrate communities, particularly decomposers and detritivores, which help maintain cave ecosystem functions (Ferreira, Prous & Martins, 2007; Ferreira, 2019). Cave-roosting bat guano can play a disproportionately important role in maintaining cavernicolous biodiversity (Gnaspini & Trajano, 2000), and this can be even more exacerbated on islands, where alternative nutrient sources are limited (Polis & Hurd, 1996). Declines in bat populations can disrupt cave ecosystems (Phelps, 2016; Frick, Kingston & Flanders, 2020). However, caves face human-induced threats like tourism, over-extraction of subterranean natural resources, vandalism and urbanisation (Furey & Racey, 2016) that can cause bats to abandon roosts, depriving caves of guano input (Sedlock & Ingle, 2010) and potentially triggering cascading effects on cavernicolous food webs (Parimuchová et al., 2021). Human-caused climate and land-use change can also alter cavernicolous community composition (Nanni et al., 2023) and, thereby, modify trophic inputs (Vaccarelli et al., 2023).

Oceanic islands can be natural laboratories for studying the impacts of human activities on biodiversity, as their geographical isolation, low functional redundancy, high endemicity, and small size amplify ecological responses to disturbances (Whittaker et al., 2017; Russell & Kueffer, 2019). Examining cave-roosting bats’ roles there can reveal how anthropogenic pressures can disrupt ecological networks (Furey & Racey, 2016; Phelps, 2016), particularly in systems with minimal interspecific interactions (Salinas-Ramos et al., 2020). One such island is Mauritius, which exemplifies oceanic islands, hosting a depauperate, but highly endemic fauna typical of volcanic islands (Cheke & Hume, 2008), and forming part of a biodiversity hotspot (Mittermeier et al., 2011). Since human settlement in 1638, habitat transformation has rapidly reduced its native vegetation twenty-fold (Hammond et al., 2015; Seetah et al., 2022), leaving highly fragmented remnants (Florens, 2013) which are today severely invaded by alien plants (Florens et al., 2016). Two bat species have already gone extinct (Cheke & Hume, 2008), leaving three surviving species, of which two are endangered (Bergmans et al., 2017; Kingston et al., 2018). The frugivorous *Pteropus niger* was subjected to repeated mass-culling campaigns (Florens, 2016; Vincenot, Florens & Kingston, 2017; Florens & Vincenot, 2018), while the lava-tube cave-roosting insectivorous bat *Mormopterus acetabulosus*, faces multiple ongoing anthropogenic threats (Bunsy et al., 2024). As the island’s only cave-roosting bat species, *M. acetabulosus* likely provides allochthonous nutrient inputs into cave ecosystems, akin to other cave bats (Pellegrini & Ferreira, 2013; Ferreira, 2019), but this potential influence remains unknown.

In this context, we studied *M. acetabulosus* as a model for insectivorous cave-roosting bats, inhabiting an island system where anthropogenic pressure on bat populations is high and increasing. This situation offers a window into what is likely to emerge in other islands as the main trends like rapid urbanisation (Koenig et al., 2025) and high human population density (United Nations, 2024) continue and bring those regions close to the situations already prevailing on Mauritius. Specifically, we investigated the role of *M. acetabulosus*’ allochthonous production in shaping cave macro-invertebrate communities. Our study thus aims to: (1) compare macro-invertebrate diversity, abundance, and community composition at the larger spatial scale of caves with and without bats; (2) compare the characteristics of macro-invertebrate fauna within caves where bat roosts are established, in relation to the distance from the roosts (before, at and beyond the roosts); and (3) assess the potential influence of the loss of *M. acetabulosus* on the cave macro-invertebrates. We predict that the presence of roosts would be a key driver of macro-invertebrate diversity, with caves containing bat colonies supporting more diverse and abundant macro-invertebrate communities than those without. Within bat-occupied caves, we expect macro-invertebrate diversity and abundance to peak at guano deposits and decline with distance from it, whereas caves without bats may exhibit weaker diversity gradients and abundance. By addressing these predictions, this study will provide insights into cave ecosystem dynamics and inform conservation strategies for threatened subterranean habitats on oceanic islands.

## Methods

### Study area

Mauritius (centred on 20°15′S and 57°35′E, 828 m a.s.l, 1,865 km^2^), is a volcanic oceanic island in the Western Indian Ocean ∼900 km east of Madagascar, formed 8.9 million years ago (Moore et al., 2011) and whose last volcanic activity dates to ∼14,000 years ago (Quidelleur & Famin, 2024). It has a mean annual temperature of 22 °C and annual rainfall ranging from 800 mm in the western lowlands to 4,000 mm in the uplands (Staub, Stevens & Waylen, 2014). The original vegetation included palm-rich woodlands, semi-dry evergreen forests, and wet-to-moist forests (Cheke & Hume, 2008; Vaughan & Wiehe, 1937). Sugarcane fields (*Saccharum officinarum*), a non-native cash crop, previously dominated the surface of Mauritius, covering 54.1% of the island, but has declined to 20.2% cover by 2023 (Mahadea-Nemdharry, Doorga & Busawon, 2025). Planted forests currently cover 22.3% of the island whereas remnants of heavily invaded native vegetation (Florens et al., 2016) cover 4.4% (Hammond et al., 2015). Other land uses include 7.1% sparse vegetation (prairies with grassland and sparse trees) and 5.5% built-up areas in 2015 (Nigel, Rughooputh & Boojhawon, 2015). Built-up areas have increased by 93%, between 2015 and 2023 to reach 10.6% today (Mahadea-Nemdharry, Doorga & Busawon, 2025). The island ranks among the most densely populated countries in Africa (615 people per km^2^; United Nations, 2024).

### Mauritian cave biodiversity

Of the 143 caves recorded on Mauritius, 130 are lava tunnels (Middleton, 1998; Middleton & Hauchler, 1998), with a combined length of approximately 14.1 km (hereafter referred to as caves). These caves are elongated, tubular formations, varying in length from seven to 1,015 m, with an average width of eight meters and height of four meters. They host colonies of two native vertebrates: the Mauritian free-tailed bat (*Mormopterus acetabulosus*) and the Mascarene Swiftlet (*Aerodramus francicus*) (Middleton & Hauchler, 1998; Wijnhorst et al., 2024), both of which contribute guano inputs that potentially sustain cavernicolous food webs. Mauritian caves support diverse, yet largely undocumented, invertebrate fauna (Middleton, 1998; Middleton & Hauchler, 1998). A subspecies of louse (*Dennyus carljonesi forresteri*), that is unique to *A. francicus* is found in Mauritian caves (Clayton, Price & Page, 1996). Previous studies have documented several invertebrates, including the only endemic Mauritian silverfish (*Lepidospora mascaraniensis*) (Mendes, 1996) and an amphipod (*Brevitalitrus strinatii*) not previously recorded in the Indian Ocean region (Stock, 1997). More species were collected in 1998, including centipedes (Scutigera), eyeless harvestmen (Opiliones), spiders (Araneae), bristletails (Thysanura), woodlice (Isopoda), myriapods (Symphyla), beetles (Coleoptera), earwigs (Dermaptera), flies (Diptera) including a wingless one, and millipedes (Diplopoda) (Humphreys, pers. comm. in Middleton, 1998). However, most of these species have not yet been identified to the species level.

### Macro-invertebrate sampling

We sampled eight caves and 137 sectors within them for macro-invertebrates by establishing sampling stations at 10% interval of the total cave length (*e.g.*, for a 100-m-long cave, the sampling interval was 10 m), starting from the cave entrance, continuing to the end of the cave (Wynne et al., 2018) (Table S1, Fig. S1). Sampling was done twice during summer (March–April 2025) and twice during winter (August–September 2025) to capture any seasonal variation in macro-invertebrate communities. We selected four caves with permanent bat colonies based on accessibility and the absence of Mascarene Swiftlet colonies to avoid additional guano input bias. Four additional caves without bat colonies were then chosen to match the bat roosting caves in length and volume while also ensuring the absence of Mascarene Swiftlets. In caves with bat colonies, transects were further divided into three zones: pre-guano (from the entrance to the guano pile edge), guano (across the guano pile), and post-guano (beyond the guano pile). At each of the sampling stations, we used three sampling methods to capture macro-invertebrates: (1) non-baited pitfall traps for ground-dwelling species; (2) timed-searches within quadrats for four minutes (Wynne et al., 2018); and (3) sticky traps to capture flying or surface-active species. Ambient cave temperature (°C) and relative humidity (%) were measured with a thermo-hygrometer (BL-20TRH Voltcraft) and light intensity level sampled with a hand-held lux meter, across each sampling stations to assess how these factors may influence macro-invertebrate abundance, distribution, and diversity. Traps remained in place for one week at each sampling across all sampling sites before retrieval.

**Figure 1:**
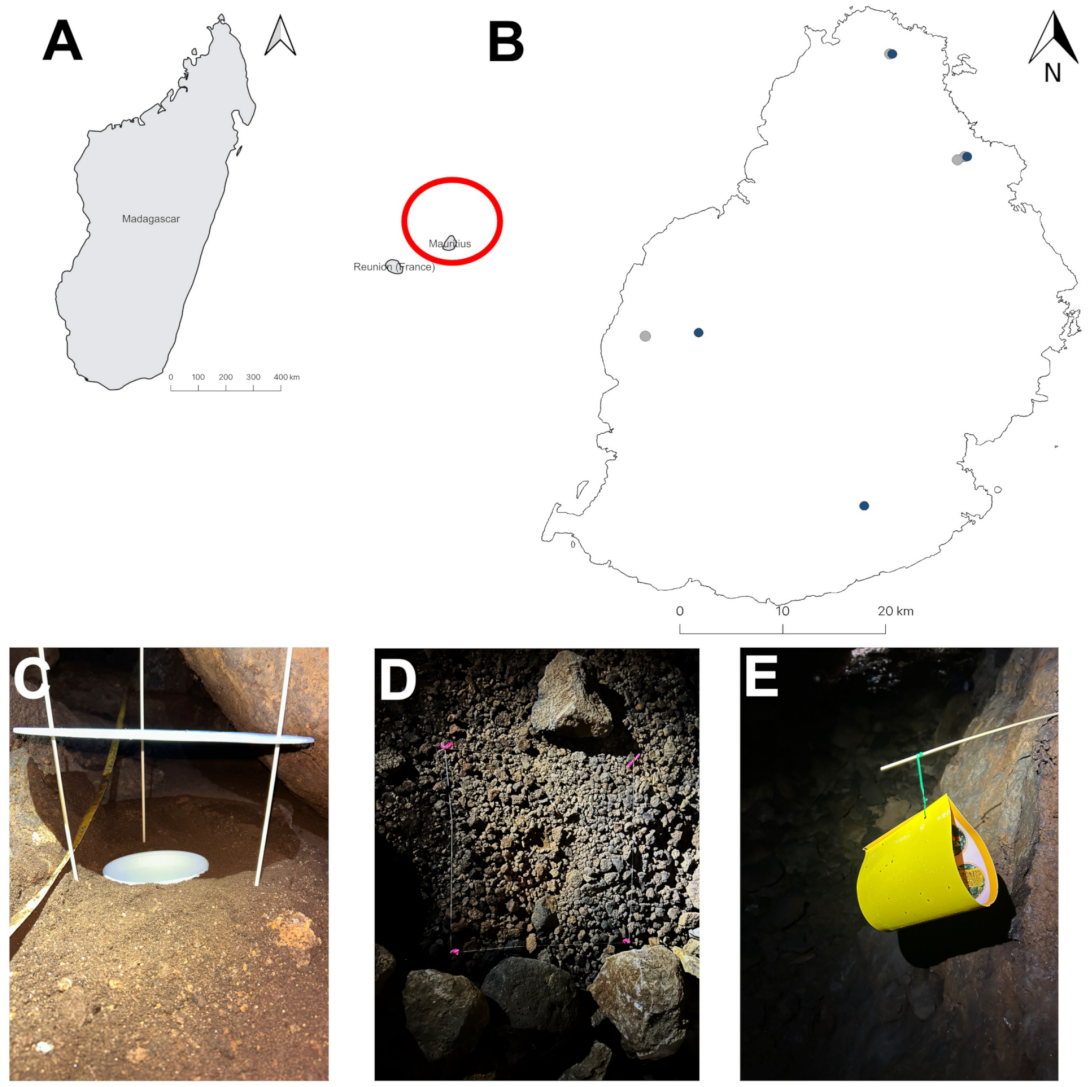
Location of sampled caves and macro-invertebrate sampling methods in Mauritius island across summer (March–April 2025) and winter (August–September 2025). (A) Mauritius (red circle) relative to Madagascar in the South-West Indian Ocean. (B) Sampled caves: Blue dots indicate caves with *Mormopterus acetabulosus*, grey dots indicate caves without bats. (C) Pitfall trap embedded flush with cave substrate, protected with a small cover to minimise debris and guano input. (D) 1 × 1 m^2^ quadrat used for systematic timed-searches (four minutes) for the collection of surface-dwelling macro-invertebrates. (E) Sticky trap deployed near cave walls to capture flying and climbing macro-invertebrates. Maps were created using QGIS (*v.* 3.16.4-Hannover). Photo credit: Yogishah Bunsy.

Pitfall traps were deployed to capture ground-dwelling and mobile macro-invertebrates. Each trap consisted of a ∼1 L nested plastic container (19 × 13 × 13 cm), embedded flush with the cave substrate to minimise avoidance (Fig. 1C). A mix of water and dish soap solution (5:1) was added to break the surface tension and prevent escape (Upton & Mantle, 2010). Where embedding was not possible, we built small ramps made of natural materials (*e.g.*, rocks, wood debris) to facilitate access. Polystyrene circular lids (∼12 cm diameter) supported by toothpicks were positioned ∼5 cm above each pitfall trap to minimise guano, water, and debris input inside the pitfall.

Live macro-invertebrates were collected within a 1 × 1 m quadrat, divided into two equal sections for systematic observation. Each quadrat was searched for four minutes (Wynne et al., 2018) and the same people made the time-searches within each cave to minimise observer bias. Surface-dwelling organisms were dislodged using a wet brush, and a pooter was used to capture smaller or mobile species. Tweezers were used to extract larger or delicate specimens. Each quadrat was carefully examined, ensuring minimal disturbance to the substrate. We also assessed the quadrat for the percentage cover of organic debris (leaves, rotten wood, roots), gravel, mud, bat guano, human-induced disturbances (*e.g.*, burnt tyres, rubbish dumping), and water bodies. Quadrat sampling was done in all cave zones to assess invertebrate diversity across different microhabitats.

Sticky traps, composed of adhesive-coated cards (∼19 × 21 cm), were deployed at each sampling station to capture flying aerial and surface-climbing macro-invertebrates. Prior to deployment, sticky trap locations were carefully assessed to prevent accidental entanglement of bats. Traps were positioned near cave walls and along passageways to maximise contact with flying and crawling arthropods. This method complemented quadrat and pitfall sampling by detecting taxa that were less likely to be captured through ground-based techniques. Macro-invertebrates were sorted by morphospecies and preserved in 95% alcohol.

### Biodiversity indices

We quantified macro-invertebrate abundance, and diversity using species richness (number of taxa) and Shannon–Wiener index (incorporating species evenness) for each sampling point (Wynne et al., 2018). Chao1 index was computed as complementary measure of richness. To identify factors influencing macro-invertebrate abundance, richness, and diversity, and to assess whether bat colony size (proxy for guano input) influenced these, we modelled each metric as a function of the same set of ecological predictors. Bat colony size was estimated by digital photography, following Bunsy et al. (2024) and Meretsky et al. (2010). Bat colony size, cave zone (pre-guano pile, at guano pile, post-guano pile), distance from the cave entrance (m), light intensity (%), ambient temperature (°C), and relative humidity (%) were included as fixed effects (Bolker et al., 2009). Cave pair was included as a random intercept to account for the paired sampling design and shared physical characteristics (length and volume) between paired cave sections (Zuur et al., 2009). Colony size was log-transformed to reduce skewness. Model validation for Poisson models showed significant over-dispersion for abundance (dispersion ratio = 90.23, Pearson’s *χ*^2^ = 50,465.52, *p* < 0.001) and richness (dispersion ratio = 1.11, Pearson’s *χ*^2^ = 613.51, *p* = 0.04). Therefore, macro-invertebrate abundance and richness were analysed using generalised linear mixed models (GLMMs), with a negative binomial distribution (Booth et al., 2003), fitted with the R package ‘glmmTMB’ *v.* 1.1.13 (Brooks et al., 2017). Shannon–Wiener diversity, treated as a continuous response variable, was analysed using linear mixed-effects model (LMM) fitted with restricted maximum likelihood (REML) using the R package ‘*lme* 4’ v 1.1-38 (Bates et al., 2015). Prior to model fitting, multicollinearity was assessed among all predictors, using Pearson correlation with a threshold of *r* > |0.7| (Dormann et al., 2013). No strong correlations were detected, and all variables were retained (Fig. S2).

Continuous predictors were standardised to improve model convergence and effect size comparison (Schielzeth, 2010). All model assumptions were evaluated using simulation-based residual diagnostics implemented in the ‘DHARMa’ *v.* 0.4.7 package (Hartig, 2022). No substantial deviations from model assumptions were detected. Model explanatory power was quantified using marginal and conditional R^2^ values used to quantify variance explained by fixed effects and by the full models, respectively (Nakagawa & Schielzeth, 2013) using the R package ‘MuMIn’ *v.* 1.48.11 (Barton & Barton, 2015). All analyses were done in R *v.* 4.5.2 (R Core Team, 2022). All mean values are presented with respective standard errors, unless specified otherwise.

### Community composition

We analysed differences in macro-invertebrate community composition using non-metric multidimensional scaling (NMDS) based on Bray-Curtis dissimilarity (Clarke, 1993), using the ‘metaMDS’ function in the ‘vegan’ *v.* 2.7-3 package in R (Oksanen et al., 2001). To test whether community composition differed between caves with and without bats, we applied permutational multivariate analysis of variance (PERMANOVA) using ‘adonis2’ function in the ‘vegan’ package in R, with Bray–Curtis dissimilarities and 999 permutations (Anderson, 2005). Prior to interpretation of the PERMANOVA results, homogeneity of multivariate dispersion between caves with bats and caves without bats was assessed using ‘betadisper’ function to ensure that detected differences reflected shifts in community composition rather than differences in within-group dispersion (Anderson, 2006). Ordination plots were used to visualise patterns of similarity in macro-invertebrate assemblages among caves with bats and caves without bats. To assess whether macro-invertebrate community composition differed among the four caves with *M. acetabulosus* colonies, a PERMANOVA (999 permutations) was performed using the adonis2 function in the vegan package (Anderson, 2005). Pairwise PERMANOVA tests were done for each cave pair to identify which caves differed significantly after applying Bonferroni corrections (Anderson, 2001; McArdle & Anderson, 2001). Homogeneity of multivariate dispersion was assessed using the ‘betadisper’ function.

### Ethics statement

Our study protocols and field study permits were approved by the National Parks and Conservation Services (NPCS) and the Forestry Services (FS) of the Ministry of Agro-Industry and Food Security. The approval references of field study permits are NP 46/3V4 from the NPCS and FD No. 971/A/III from the FS.

## Results

### Macro-invertebrates abundance, richness, and diversity

**Figure 2:**
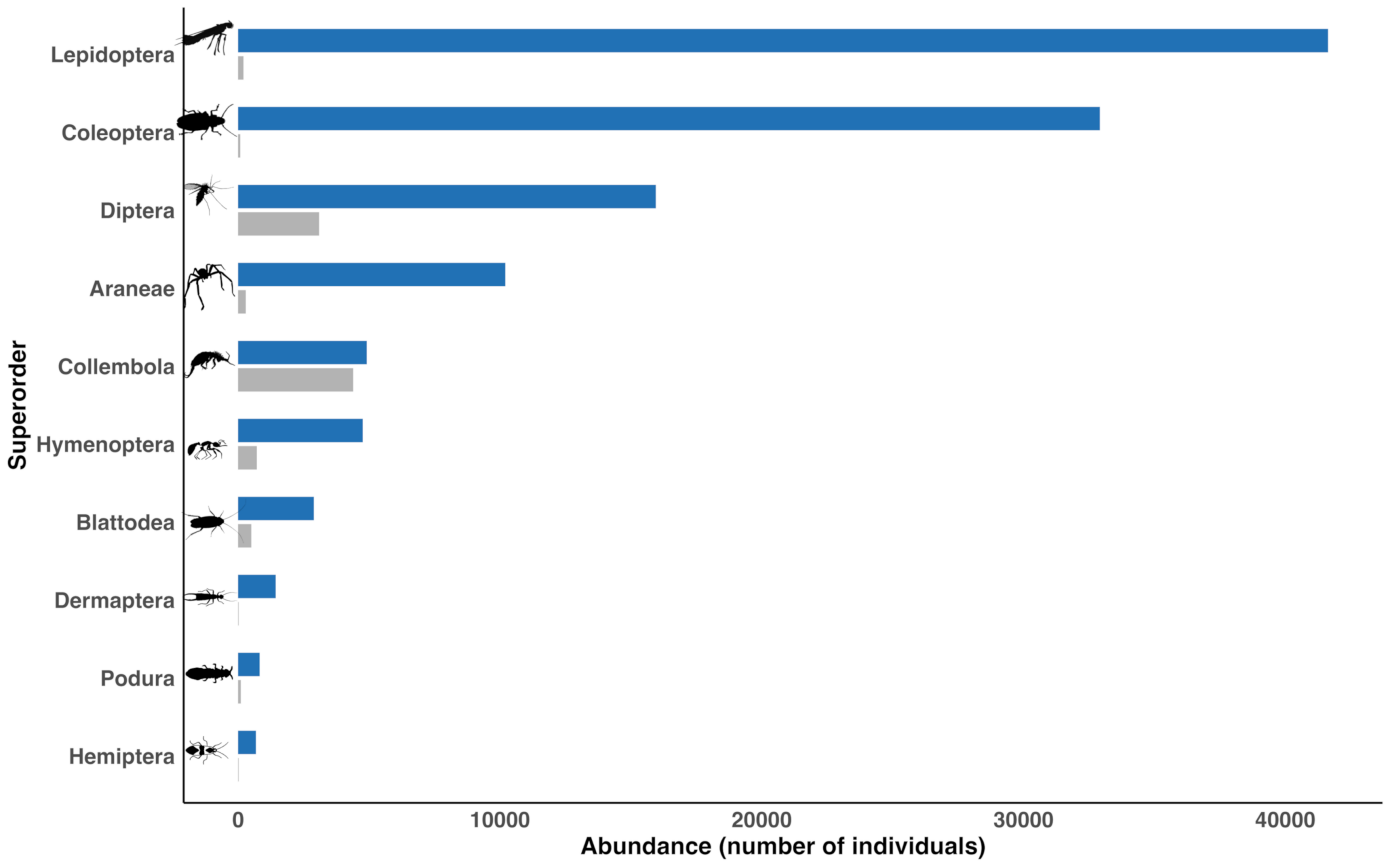
Abundance of the top ten macro-invertebrate superorders in sampled caves in Mauritius island with (blue) and without (grey) colonies of the bat, *Mormopterus acetabulosus*. Superorders are arranged from lowest to highest abundance in caves with *M. acetabulosus* colonies. All differences in abundances of macro-invertebrate orders presented are significant at *p* < 0.001. Silhouette images of macro-invertebrates were designed by: https://www.phylopic.org/.

**Figure 3:**
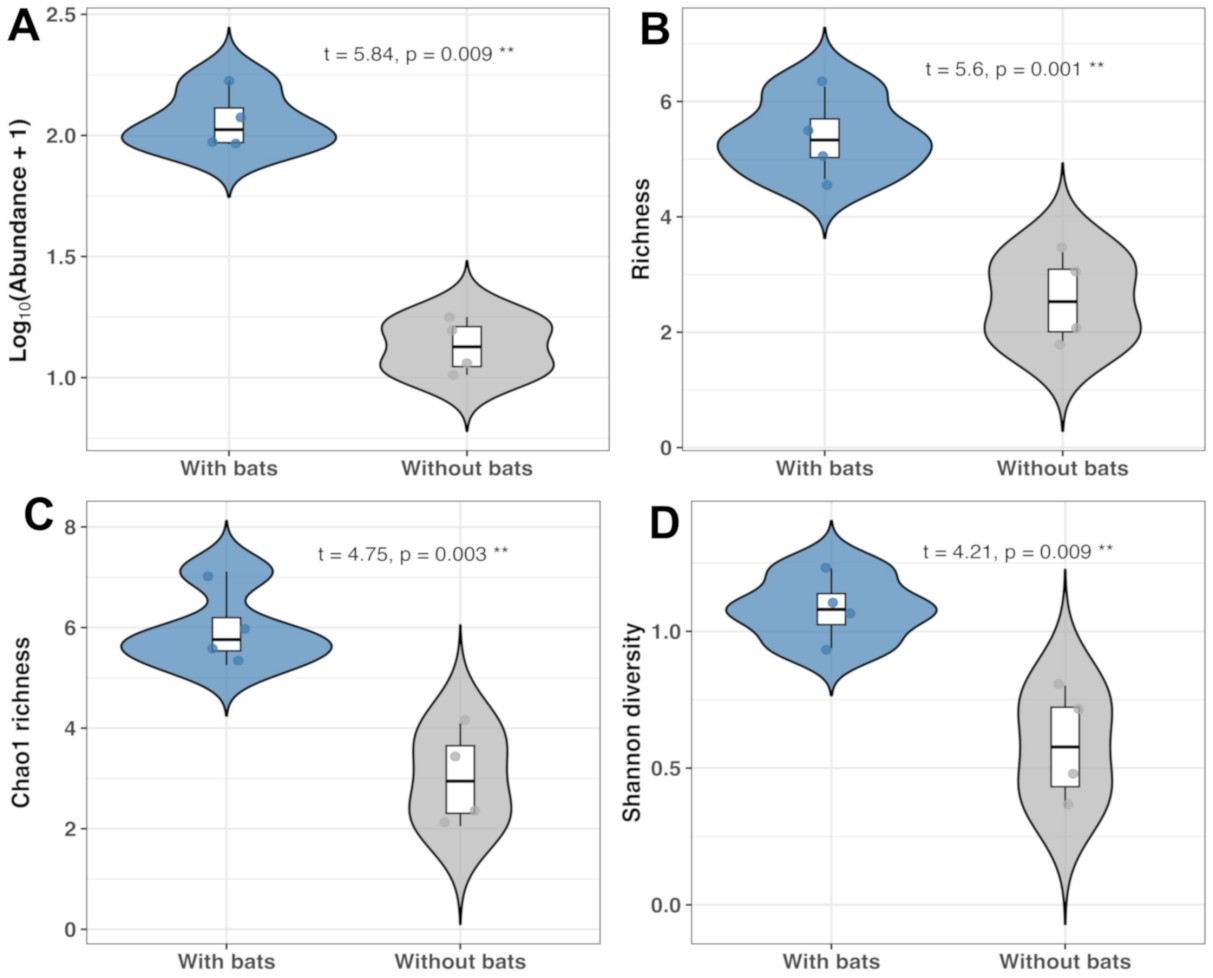
Comparison of macro-invertebrate community metrics between caves in Mauritius island with (blue) and without (grey) colonies of the bat *Mormopterus acetabulosus*. Violin plots show the distribution of (A) log_10_-transformed macro-invertebrate abundance (+1), (B) species richness, (C) Chao1 estimated richness, and (D) Shannon diversity. Boxplots embedded within violins indicate the median and interquartile range, with individual points representing cave-level values. Statistical comparisons between cave categories were performed using two-sample t-tests, with test statistics (t), *p*-values, and significance levels (*p* < 0.05 *, < 0.01 **, < 0.001 ***) displayed on each panel.

Across all eight sampling sites (137 sampling sectors were each surveyed four times, across two seasons), 127,170 macro-invertebrates were recorded (caves with *M. acetabulosus* colonies: 117,530 (94.4%), caves without colonies: 9,640 (7.6%), representing 86 morphospecies (Fig. S3). The 10 most abundant super-orders in caves with *M. acetabulosus* colonies were Lepidoptera (35.4%), Coleoptera (28.0%), Diptera (13.6%), Araneae (8.7%), Collembola (4.2%), Hymenoptera (4.1%), Blattodea (2.5%), Dermaptera (1.2%), Podura (0.7%), and Hemiptera (0.6%) (Fig. 2). Correspondingly, for caves without bats, they were Collembola (44.1%), Diptera (33.4%), Hymenoptera (7.5%), Blattodea (5.2%), Araneae (3.0%), Lepidoptera (2.1%), Coleoptera (0.9%), Podura (0.9%), Dermaptera (0.2%), and Hemiptera (0.2%). Macro-invertebrate abundance was significantly higher in caves with bats than where bats are absent (*t* = 5.84, *p* = 0.009). Species richness was also greater in caves with bat colonies compared to caves without bat colonies (*t* = 5.6, *p* = 0.001). Chao1 estimates followed the same trend, with higher values in caves with bats than in caves without bats (*t* = 4.75, *p* = 0.003). Shannon diversity was significantly higher in caves with bats relative to caves without bats (*t* = 4.21, *p* = 0.009) (Fig. 3, Table S2).

Species richness was significantly higher in summer (March–April, wet season) compared to winter (August–September, dry season) in caves without bat colonies (*z* = 6.85, *p* < 0.001). Shannon diversity followed the same trend, with higher richness in summer compared to winter in caves without bats (*t* = 11.47, *p* < 0.001). No seasonal difference was observed in species richness and Shannon diversity in caves where bats are present (t = −0.42, *p* = 0.672). No significant seasonal effect on macro-invertebrate abundance was observed either in caves with bats (z = −1.38, *p* = 0.168) or caves without bats (*z* = 1.14, *p* = 0.255) (Fig. S4, Table S3).

### Drivers of diversity metrics

Macro-invertebrate abundance, richness, and diversity were positively associated with bat colony size (abundance GLMM *β* = 0.35 ± 0.05, *p* < 0.001, richness: *β* = 0.20 ± 0.03, *p* < 0.001, diversity LMM *β* = 0.06 ± 0.01, *p* < 0.001), and were highest in guano zones, differing significantly from other cave zones (pre-guano, post-guano and no-guano) (Tables 1–3). Distance from the cave entrance was negatively associated with abundance, richness, and diversity (abundance: *β* = −0.09 ± 0.02, *p* < 0.001; richness: *β* = −0.12 ± 0.02, *p* < 0.001; diversity: *β* = −0.02 ± 0.004, *p* < 0.001). Light intensity was also positively associated with macro-invertebrate abundance, richness, and diversity (abundance: *β* = 0.25 ± 0.03, *p* < 0.001; richness: *β* = 0.15 ± 0.02, *p* < 0.001; diversity: *β* = 0.02  ± 0.01, *p* < 0.001, Fig. S5). Relative humidity was positively associated with diversity (*β* = 0.01 ± 0.01, *p* = 0.016) and negatively associated with abundance (*β* = −0.07 ± 0.03, *p* = 0.010) and richness (*β* = −0.06 ± 0.01, *p* = 0.001). Temperature (^∘^C) showed no association with the three metrics.

**Table 1:** Parameter estimates of the Generalised Linear Mixed Model (GLMM) explaining macro-invertebrate abundance across zones in caves in Mauritius island with colonies of the bat *Mormopterus acetabulosus* (Guano zone as reference). Fixed effects include log-transformed bat colony size, cave zone, distance from the cave entrance (scaled, m), light intensity (scaled, %), ambient temperature (scaled, °C), and relative humidity (scaled, %). Estimates are presented with standard error (SE), Z-value, and associated *p*-values. Statistically significant variables are in bold (*N* = 1, 692, Marginal *R*^2^ = 0.78, Conditional *R*^2^ = 0.79). Model residuals showed no evidence of overdispersion (dispersion test *p* = 0.032), indicating that model assumptions were reasonably met.

	**Estimate**	**SE**	**Z value**	***P*-value**
(Intercept)	7.20	0.06	116.07	**<0**.**001**
Colony size (log)	0.35	0.05	6.46	**<0**.**001**
No guano zone (guano zone reference)	−3.68	0.07	−53.59	**<0**.**001**
Pre guano zone (guano zone reference)	−2.20	0.07	−29.91	**<0**.**001**
Post guano zone (guano zone reference)	−2.84	0.08	−37.79	**<0**.**001**
Distance (m; scaled)	−0.09	0.02	−3.75	**<0**.**001**
Temperature (°C; scaled)	−0.02	0.02	−1.13	0.260
Light intensity (%; scaled)	0.25	0.03	10.35	**<0**.**001**
Humidity (%; scaled)	−0.07	0.03	−2.52	**0**.**010**

**Table 2:** Parameter estimates of the Generalised Linear Mixed Model (GLMM) explaining macro-invertebrate richness across zones in caves in Mauritius island with colonies of the bat *Mormopterus acetabulosus* (Guano zone as reference). Fixed effects include log-transformed bat colony size, cave zone, distance from the cave entrance (scaled, m), light intensity (scaled, %), ambient temperature (scaled, °C), and relative humidity (scaled, %). Estimates are presented with standard error (SE), Z-value, and associated *p*-values. Statistically significant variables are in bold (*N* = 1, 692; Marginal *R*^2^ = 0.55, Conditional *R*^2^ = 0.59). Model residuals showed no evidence of overdispersion (dispersion test *p* = 0.376), indicating that model assumptions were reasonably met.

	**Estimate**	**SE**	**Z value**	***P*-value**
(Intercept)	2.66	0.07	39.13	**<0**.**001**
Colony size (log)	0.20	0.03	7.69	**<0**.**001**
No guano zone (guano zone reference)	−0.92	0.04	−25.35	**<0**.**001**
Pre guano zone (guano zone reference)	−0.31	0.04	−8.20	**<0**.**001**
Post guano zone (guano zone reference)	−0.52	0.04	−12.35	**<0**.**001**
Distance (m; scaled)	−0.12	0.02	−7.47	**<0**.**001**
Temperature (°C; scaled)	−0.01	0.02	−0.87	0.386
Light intensity (%; scaled)	0.15	0.02	10.01	**<0**.**001**
Humidity (%; scaled)	−0.06	0.02	−3.36	**0**.**001**

**Table 3:** Parameter estimates of the Linear Mixed Model (LMM) explaining Shannon diversity of macro-invertebrates across zones in caves hosting colonies of *Mormopterus acetabulosus* (Guano zone as reference). Fixed effects include log-transformed bat colony size, cave zone, distance from the cave entrance (scaled, m), light intensity (scaled, %), ambient temperature (scaled, °C), and relative humidity (scaled, %). Estimates are presented with standard error (SE), t-value, and associated *p*-values. Statistically significant variables are in bold (*N* = 1,692, Marginal *R*^2^ = 0.47, Conditional *R*^2^ = 0.93). Model residuals showed no evidence of overdispersion (dispersion test *p* = 0.792), indicating that model assumptions were reasonably met. The No Guano zone represents caves without bat colonies of *M. acetabulosus*.

	**Estimate**	**SE**	**Z value**	***P*-value**
(Intercept)	2.24	0.15	15.03	**<0**.**001**
Colony size (log)	0.05	0.01	4.71	**<0**.**001**
No guano zone (guano zone reference)	−0.54	0.01	−43.74	**<0**.**001**
Pre guano zone (guano zone reference)	0.05	0.01	3.71	**<0**.**001**
Post guano zone (guano zone reference)	0.10	0.01	7.01	**<0**.**001**
Distance (m; scaled)	−0.02	0.004	−3.98	**<0**.**001**
Temperature (°C; scaled)	−0.01	0.004	−1.80	0.072
Light intensity (%; scaled)	0.02	0.01	4.88	**<0**.**001**
Humidity (%; scaled)	0.01	0.01	2.40	**0**.**016**

### Community composition

**Figure 4:**
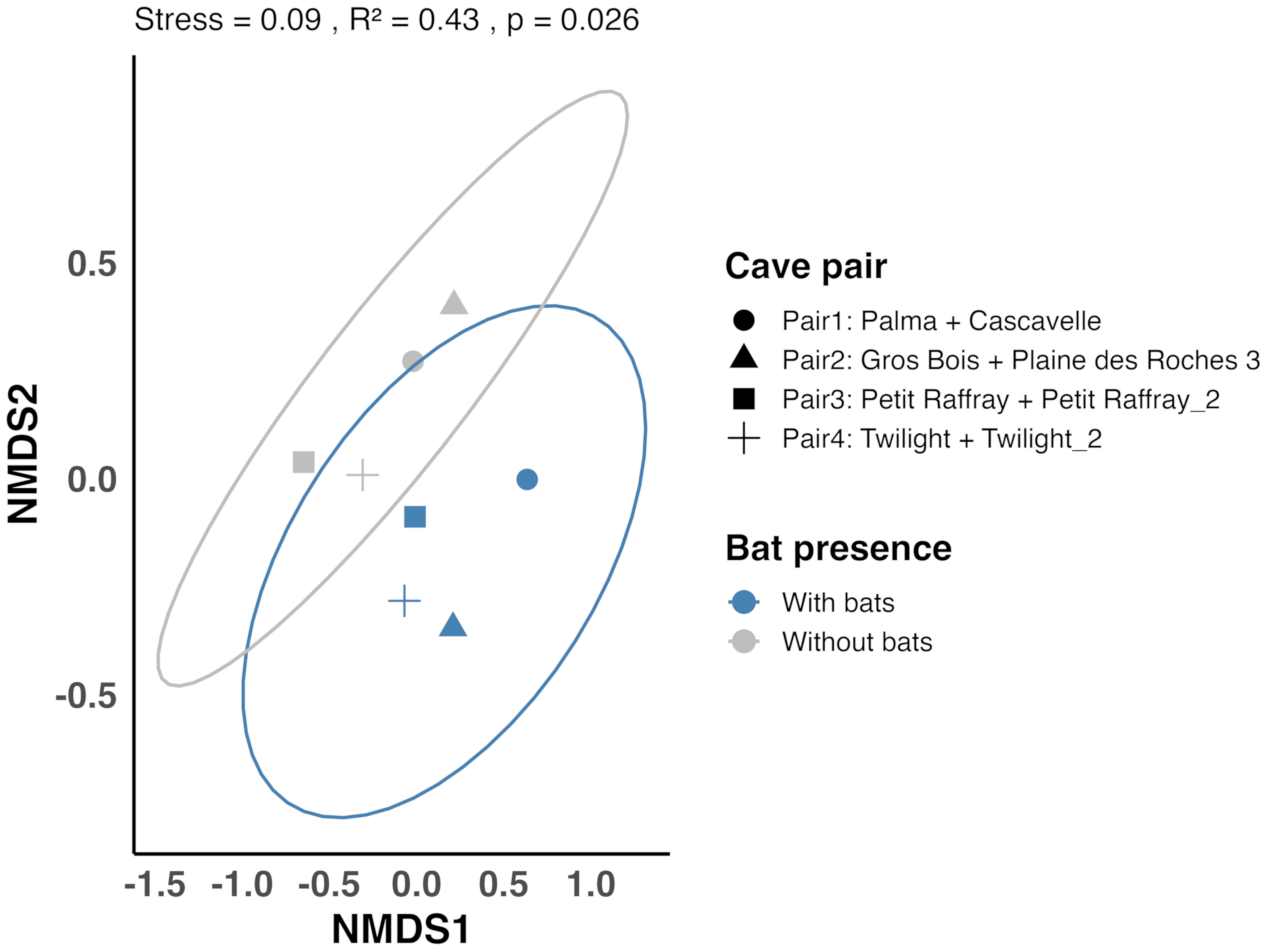
Non-metric multidimensional scaling (NMDS) ordination based on Bray–Curtis dissimilarities between caves in Mauritius island with (blue) and without (grey) colonies of the bat *Mormopterus acetabulosus*. Each point represents a cave, with symbol shapes indicating paired caves and colours denoting bat presence. Ellipses represent 95% confidence intervals around group centroids. The ordination shows good fit (stress = 0.09), and PERMANOVA indicates a significant difference in community composition between cave categories (*R*^2^ = 0.43, *p* = 0.025).

Beta diversity analyses based on non-metric multidimensional scaling (NMDS) ordination showed a clear separation of macro-invertebrate communities according to bat presence (stress = 0.090, Fig. 4). PERMANOVA indicated that bat presence explained 43% of the variation in community composition across caves (*R*^2^ = 0.43, F_1_,_6_ = 4.46, *p* = 0.025). Homogeneity of multivariate dispersion did not differ between groups (*F* = 0.73, *p* = 0.43), indicating that the observed differences reflect true changes in community composition rather than differences in variance. Pairwise PERMANOVA showed that macro-invertebrate communities differed significantly among most sampled caves, with effect sizes ranging from moderate to high (R^2^ = 0.040–0.123, Fig. S6). Notably, all caves hosting *M. acetabulosus* colonies (Gros Bois, Palma, Petit Raffray and Twilight) differed significantly from each other. The strongest differentiation was between Gros Bois and Petit Raffray (*R*^2^ = 0.082, p_adj = 0.028), followed by Gros Bois and Palma (*R*^2^ = 0.064, p_adj = 0.028), Petit Raffray and Twilight (*R*^2^ = 0.062, p_adj = 0.028), and Gros Bois and Twilight (*R*^2^ = 0.060, p_adj = 0.028). Significant differences also occurred between bat-occupied caves and unoccupied caves, with Petit Raffray (with bat colonies) differing from these unoccupied caves: Twilight 2 (*R*^2^ = 0.098, p_adj = 0.028), Petit Raffray 2 (*R*^2^ = 0.084, p_adj = 0.028), and Plaine des Roches 3 (*R*^2^ = 0.081, p_adj = 0.028). Twilight (with bat colonies) differed from Petit Raffray_2 (*R*^2^ = 0.077, p_adj = 0.028) and Plaine des Roches 3 (*R*^2^ = 0.069, p_adj = 0.028).

Bray–Curtis dissimilarity between surveyed caves showed little change with geographic distance across all sites (linear model slope = −0.0032, *R*^2^ = 0.07; Mantel r = −0.21, *p* = 0.87; Fig. S7). No significant difference was observed among bat-occupied caves (slope = −0.0071, *R*^2^ = 0.18; Mantel r = −0.49, *p* = 0.88). A comparable weak trend occurred in caves without bats (slope = −0.0042, *R*^2^ = 0.32; Mantel r = −0.26, *p* = 0.75).

## Discussion

### Ecological implications

Colonies of *M. acetabulosus* strongly influence macro-invertebrate communities of lava-tube caves on oceanic island Mauritius. Caves hosting *M. acetabulosus* colonies had substantially higher macro-invertebrate abundance, species richness, and diversity compared to caves similar in structure and location but without bats. These patterns suggest that the guano brought by *M. acetabulosus* increases the diversity of cavernicolous arthropod communities. In caves where bat colonies roost, they are responsible for major allochthonous production mainly in the form of the guano piles that form beneath the mostly fixed-position roosts. As evidenced elsewhere, such allochthonous production contrasts much with sections of the same caves further away from the roosts, resulting in a paucity of organic matter which in turn supports low macro-invertebrate richness and diversity (Jaffe et al., 2016; Howarth, Ferreira & Mammola, 2025). Guano inputs are known to support decomposers and detritivores, which in turn support predator and omnivore food chains and webs (Dainelli et al., 2025; Ganem et al., 2026) and the same is now being confirmed and quantified for the oceanic volcanic island of Mauritius and its sole species of cave-roosting bats. While subterranean biodiversity has been documented across mainland systems (Ferreira, Prous & Martins, 2007; da Rocha Melo, Ferreira & Silva, 2025), substantial knowledge gaps remain at the regional (Oliveira et al., 2025) and global scales (Naˇpaˇruş-Aljančič et al., 2026), and comparable studies on isolated oceanic islands remain limited.

The absence of seasonal variation in macro-invertebrate richness and diversity in bat-occupied caves compared to a significant wet-season peak in unoccupied caves, reinforces the notion that guano acts as a stable allochthonous input that decouples invertebrate communities from external environmental conditions (Gnaspini & Trajano, 2000; Iskali & Zhang, 2015). The guano, therefore, provides a buffering effect that is contingent with bat roosts as caves lacking guano subsidies likely rely on seasonally variable inputs, like rainfall-mediated influx of organic matter (Silva et al., 2012) to sustain their invertebrate communities.

Caves hosting roosts of *M. acetabulosus* show marked variability of macro-invertebrate abundance, richness, and diversity that peak around where the bat guano piles form, further stressing the importance of the guano as the main factor influencing the distribution and composition of the macro-invertebrate communities. This aligns with the resource subsidy hypothesis (Leroux & Loreau, 2008), where organic inputs from guano often sustain subterranean food webs (Schneider, Christman & Fagan, 2011; Ferreira, 2019). Abundance and richness of macro-invertebrates also dropped with distance from the cave entrance (Figs. S8, S9), likely reflecting reduced nutrient input with distance from the exterior of the caves (Culver & Pipan, 2018; Culver & Pipan, 2019), aligning with other studies whereby distance from the cave entrance is a significant factor, leading to lower invertebrate richness and diversity further inside the cave (da Rocha Melo, Ferreira & Silva, 2025). Light intensity positively influenced diversity and abundance of macro-invertebrates, by likely increasing habitat heterogeneity, resource availability (through a degree of autochthonous production), and supporting light-tolerant surface taxa near cave entrances (Fei et al., 2024; Reis-Venâncio et al., 2024). Relative humidity reduced macro-invertebrate abundance and richness but increased overall diversity, suggesting that humidity alone does not limit cavernicolous macro-invertebrate communities. Temperature had no effect on the three metrics, likely due to the low thermal variability typically reported in caves (Medina et al., 2023).

The observed separation of macro-invertebrate assemblages between caves with and without *M. acetabulosus* colonies reinforces the notion that bat colonies represent a key ecological driver of cave macro-invertebrate communities (Ferreira & Martins, 1999; Ferreira, Prous & Martins, 2007; Dainelli et al., 2025). Indeed, bat-derived guano can re-structure trophic pathways by supporting detritivore assemblages and their associated predators, thereby influencing invertebrate community composition (Parimuchová et al., 2021). However, the significant dissimilarity among caves hosting *M. acetabulosus* colonies suggests that their macro-invertebrate communities do not converge towards a uniform assemblage. Instead, each cave supports a distinct community, likely reflecting differences in bat colony size, microclimatic conditions, chance colonisation of the island-like features that caves represent within the matrix of terrestrial ecosystems, and cave morphology that alters resource distribution and habitat heterogeneity. Such patterns are consistent with the view of caves as ecological “islands”, where isolated subterranean habitats support distinct communities that are structured by local environmental conditions. Previous studies have indeed shown that cave biodiversity is strongly influenced by habitat characteristics like cave size, entrance structure, and internal development, which affect resource availability (Brunet & Medellín, 2001; Culver & Pipan, 2019).

### Conservation implications

The lower richness and abundance of macro-invertebrates, characteristic of caves lacking bat roosts, suggests that communities associated with *M. acetabulosus* roosts may shift towards similar ones to those observed in caves without bat colonies should the species disappear from these caves. The seasonal buffering conferred by the bat guano suggests that losing bat roosts would not only reduce macro-invertebrate diversity to levels currently observed in unoccupied caves, but would additionally expose those communities to seasonal fluctuations in richness and diversity. The existence of cave-specialists and island endemic macro-invertebrates (Mendes, 1996; Stock, 1997) suggest that the eventual local extinction of bat roosts would potentially trigger extinction cascades which may be of local (for non-endemic macro-invertebrate taxa) to global nature (for endemic macro-invertebrate taxa). The possibility of losing roosts of the endemic and Endangered bat, *M. acetabulosus*, is real given the current anthropogenic pressures besetting the species, several of which are inexorably worsening (Hammond et al., 2015; Bunsy et al., 2024; Y Bunsy et al., 2024, unpublished data). Since larger colonies that form larger guano piles support more diverse and abundant macro-invertebrates, it would also be advisable to address any roost decline to stabilise existing colonies, and, where possible, increase roost size by improving land-use practices, reduce pesticide uses (Kahnonitch, Lubin & Korine, 2018), and providing water sources near roosts (Straka et al., 2020). The primary pressures on *M. acetabulosus* colonies include intentional cave destruction (complete loss of bat colonies), fires underneath roosts, and ongoing pollution (Bergmans et al., 2017; Bunsy et al., 2024). Mitigating these pressures would require targeted, evidence-based management actions like legal protection and physical gating of bat-occupied cave entrances, regulate access during sensitive periods (breeding season), engagement with hiking guides, and systematic monitoring of intervention efficacy to guide adaptive management (Meierhofer et al., 2024). Finally, even if bat roosts are maintaining themselves, it would not be advisable to authorise extraction of guano because of the detrimental effect that this would almost certainly have on the macro-invertebrate fauna documented here, particularly because *M. acetabulosus* is the sole species roosting in caves on the island and it is currently endangered, which means that its population, hence the guano that it produces, is already lower than historic, pre-anthropisation rates.

The dissimilar macro-invertebrate communities between the different caves hosting roosts of *M. acetabulosus* implies that biodiversity loss among macro-invertebrates would likely have already happened following the reduction of roost sizes or their loss (Bunsy et al., 2024). This would have likely occurred in the process of the bat species becoming highly threatened with extinction, since it is classified as endangered on the Red List of the International Union for Conservation of Nature (Bergmans et al., 2017). This situation is concerning from a conservation point of view because of the current lack of thorough taxonomic documentation of cavernicolous macro-invertebrates on the island. It is therefore plausible or even likely that a number of cavernicolous macro-invertebrate species have not yet been described and could have already disappeared or are disappearing because of local extinctions of bat roosts or the decline of the bat populations since human colonisation. Such ‘dark extinction’ of previously unknown macro-invertebrate species has been reported recently in Mauritius, albeit outside of caves (Gerlach, Florens & Griffiths, 2025). Currently, the so far understudied cavernicolous biota is known to host a subspecies of louse (*Dennyus carljonesi forresteri*) (Clayton, Price & Page, 1996), a silverfish (*Lepidospora mascaraniensis*) (Mendes, 1996) and an amphipod (*Brevitalitrus strinatii*) (Stock, 1997). In this context, it is important to taxonomically examine cavernicolous biodiversity in Mauritius for important species (*e.g.*, endemics), the presence of which can then be used to further guide prioritisation of caves for protection and conservation management. Meanwhile, a maximum of caves should be afforded protection, while prioritising those with allochthonous input by cave-roosting bats.

Ultimately, as the island’s cave habitats continue to sustain direct threats (Bunsy et al., 2024) and indirect pressures like encroaching urbanisation set to drive a reduction in the foraging habitat quality for the bats (Y Bunsy et al., 2024, unpublished data), it appears imperative to start with affording the currently missing legal protection to both the Endangered bat and their specialised cave roost sites (Bunsy et al., 2024). However, in Mauritius, protection alone is known to be insufficient to effectively conserve biodiversity, be it at species level (Florens, 2015; Florens, 2016) or at habitat level (Bissessur, Baider & Florens, 2017; Florens et al., 2017). Therefore, active conservation management is warranted to stem and reverse the ongoing threats and to restore degraded roosting sites and habitats of the bats. The legal protection and active management of existing roosts must constitute the primary conservation priority, along with stopping anthropogenic threats (illegal dumping, poaching, and fires) at candidate unoccupied caves (Bunsy et al., 2024) as prerequisite steps (Meierhofer et al., 2024). Only once these conditions are met should the establishment of new colonies in suitable lava caves be pursued as a complementary strategy to reduce the risks of extinction of the endangered bat and of the cave biota dependent on the allochthonous production that it vectors into these caves. While the establishment of new bat roosts may hold some challenges, once this would have happened, the re-introduction or introduction of cavernicolous biota associated with the guano could contribute to restoring cave communities to a state closer to their original biota. The establishment of such new roosts and attendant guano-dependant biota would serve as elevated insurance against extinction risks of both the bats and the cavernicolous macro-invertebrate and other life forms associated with guano piles, while extending the insect pest control service of the bat.

## Conclusion

This study shows that colonies of an island’s sole cave-roosting insectivorous bat, (*Mormopterus acetabulosus*), play a key ecological role in structuring cavernicolous biodiversity through allochthonous nutrient inputs. Additionally, findings show that each cave hosts a distinct macro-invertebrate assemblage, and the decline or disappearance of a bat roost could result in the loss of biodiversity that potentially include endemic species. Our results, therefore confirm that bat colonies and their guano inputs shape macro-invertebrate abundance, richness and diversity within a ‘double islandness system’ (small isolated cave habitats within a small isolated oceanic island). This can provide an entry point for the study and conservation of invertebrates—a group that remains poorly documented in Mauritius (Motala et al., 2007). Our study provides additional support to reinforce the need for legally protecting existing bat roosts, as well as facilitating the establishment of colonies in currently unoccupied caves which could buffer potential extinction risks.

##  Supplemental Information

10.7717/peerj.21622/supp-1 Supplemental Information 1 Map and schematic representation of the sampling design used in 2025 to sample caves in Mauritius island with and without colonies of the bat *Mormopterus acetabulosus* “Twilight Cavern” (Plaine des Roches cave area, north-east Mauritius), adapted from Middleton (1998), is used as an example of a sampled cave. (a) Example of a cave plan with the surveyed extent and spatial configuration of the system. (b) Schematic representation of the adapted macro-invertebrate sampling methodology along the sampled caves’ horizontal lengths. Sampling stations were established at 10% interval of the total cave length. Sampling progressed from the cave entrance towards the interior until the end of the cave passage. At each station, three complementary methods were applied: SM1: time-constrained searches (4 minutes) within a 1 × 1 m quadrat; SM2: non-baited pitfall traps; and SM3: sticky traps. All icons designed by: https://www.freepik.com. Photo credit: Daphney Dupré.

10.7717/peerj.21622/supp-2 Supplemental Information 2 Pearson correlation matrix of predictor variables used in the Generalised Linear Mixed Model (GLMMS) and Linear Mixed Model (LMM) analyses of macro-invertebrate communities in caves in Mauritius island The matrix shows pairwise correlations between ecological predictors, including bat colony size, cave zone (pre-guano, at guano, post-guano), distance from cave entrance (m), light intensity (%), ambient temperature (C), and relative humidity (%). Correlation coefficients (r) are shown inside each cell, with blue shades indicating positive correlations and red shades indicating negative correlations. Correlations exceeding —r— > 0.7, indicative of potential multicollinearity, were used to guide variable selection for the global models (Dormann et al., 2013). No strong correlations were detected in our dataset, and all predictors were retained for analyses of macro-invertebrate abundance, species richness, Shannon diversity.

10.7717/peerj.21622/supp-3 Supplemental Information 3 Examples of macro-invertebrates recorded in eight sampled lava caves of Mauritius island during surveys. Photographs illustrate examples of the taxonomic diversity of macro-invertebrates Panels show orders: Araneae (a-d, h, t), Blattodea (f), Collembola (k, r), Coleoptera (g, m, u, w, x), (Diptera (v), Hemiptera (o), Hymenoptera (l, n, p, q), Pseudoscorpiones (j), Polydesmida (i), Scolopendromorpha (e), and Zygentoma (s). Photo credits: Pierre-Henry Cressent (a-d, g, n), Nicolas Huet (i), and Yogishah Bunsy (e, f, h, j-m, o-x).-m, o-x).

10.7717/peerj.21622/supp-4 Supplemental Information 4 Seasonal variation in macro-invertebrate assemblages in sampled caves in Mauritius island with (blue) and without (grey) colonies of the bat, Mormopterus acetabulosus Panels show (a) abundance (log scale), (b) richness, and (c) Shannon diversity across the wet (summer; March-April) and dry (winter; August-September) seasons (mean ± SE). Lines connect seasonal means within each cave category. Statistical comparisons between seasons within each category are indicated above brackets: ns = not significant, *** = *p* < 0.001.

10.7717/peerj.21622/supp-5 Supplemental Information 5 Relationship between distance (m) from cave entrance, light intensity (%), and macro-invertebrate community metrics (abundance, richness, and diversity) across the eight surveyed caves in Mauritius island Scatterplots show changes in macro-invertebrate abundance (in blue lines), richness (green lines), and diversity (yellow lines) along distance from the cave entrance. Points represent observed values and solid lines represent smoothed trends. Dashed black line shows the decline in light intensity with increasing distance from the cave entrance. Light intensity decreases along distance, approaching zero values deeper in caves.

10.7717/peerj.21622/supp-6 Supplemental Information 6 Pairwise beta diversity (*R*^2^) of macro-invertebrate assemblages among sampled caves in Mauritius island (a) Heatmap shows pairwise comparisons of community composition between caves with *Mormopterus acetabulosus* colonies, where rows and columns represent individual caves and cell values indicate the coefficient of determination (*R*^2^). Higher *R*^2^ values (darker blue) reflect greater differentiation in assemblage structure between cave pairs, whereas lower values (lighter colours) indicate more similar communities. Asterisks (*) denote statistically significant differences between caves based on pairwise PERMANOVA tests with adjusted p-values (p_adj_ < 0.01). (b) Dendrogram illustrating the similarity in community composition among caves, constructed using hierarchical clustering of pairwise PERMANOVA *R*^2^ values. Caves that cluster more closely together have more similar assemblage structure, whereas those separated by longer branch lengths are more strongly differentiated. Photo credit: Daphney Dupré.

10.7717/peerj.21622/supp-7 Supplemental Information 7 Relationship between geographic distance and macro-invertebrate community dissimilarity among caves Bray–Curtis dissimilarity values were calculated from macro-invertebrate assemblages and plotted against geographic distance between cave pairs, sampled in Mauritius island. Panels show results for (a) all caves combined, (b) caves hosting Mormopterus acetabulosus colonies, and (c) caves without bat colonies. Each point represents a pairwise comparison between two caves. Blue lines represent linear model fits illustrating the trend between geographic distance (km) and community dissimilarity. Mantel tests were used to assess the correlation between geographic distance and Bray–Curtis dissimilarity matrices, with Mantel’s r and associated p-values shown in each panel.

10.7717/peerj.21622/supp-8 Supplemental Information 8 Variation in macro-invertebrate abundance along distance gradients from cave entrances in paired sampled caves, in Mauritius island with (blue) and without (grey) colonies of the bat, Mormopterus acetabulosus The x-axis represents distance from the cave entrance (m), and the y-axis shows macro-invertebrate abundance recorded at each sampling point. Vertical dashed lines indicate the approximate location of bat roosting zones within caves with colonies. Circles highlight major peaks in macro-invertebrate abundance associated with these roosting areas. Shaded areas in Pair 4 indicate sections of the cave where bat roosts and guano accumulation are sequential. Circle size reflects the relative size of *M. acetabulosus* colonies at those locations, with larger circles indicating larger aggregations of bats.

10.7717/peerj.21622/supp-9 Supplemental Information 9 Variation in macro-invertebrate richness along distance gradients from cave entrances in paired sampled caves in Mauritius island with (blue) and without (grey) colonies of the bat, Mormopterus acetabulosus Each panel represents a cave pair, with caves hosting bat colonies shown in blue and caves without colonies shown in grey. The x-axis represents distance from the cave entrance (m), and the y-axis shows macro-invertebrate richness recorded at each sampling point. Vertical dashed lines indicate the approximate location of bat roosting zones within caves hosting colonies. Circle size reflects the relative size of M. acetabulosus colonies at those locations, with larger circles indicating larger aggregations of bats. Shaded areas in Pair 4 indicate sections of the cave where bat roosting activity and guano accumulation were sequential.

10.7717/peerj.21622/supp-10 Supplemental Information 10 Main characteristics of the four sampled caves in Mauritius island with roosts of the bat, *Mormopterus acetabulosus*, corresponding physical traits, and guano sizes Latitude and longitude of roosting localities are not provided to avoid elevating risks to the target species. Ma: Millions of years. Names between inverted commas refer to that used in (Middleton & Hauchler, 1998). The estimated guano area m 2) was estimated by multiplying the largest length (L) of the guano pile by the largest width (W).

10.7717/peerj.21622/supp-11 Supplemental Information 11 Mean ± standard error (SE) of macro-invertebrate abundance, richness, and diversity indices per sample in caves with *Mormopterus acetabulosus*’ guano input and in caves without guano input

10.7717/peerj.21622/supp-12 Supplemental Information 12 Estimated marginal means ± standard error (SE) of macro-invertebrate abundance, species richness, and Shannon diversity across seasons (Wet: March–April; Dry: August–September) in samples caves in Mauritius island with and without colonies of the bats Seasonal contrasts (wet–dry) are shown for each cave category, with corresponding test statistics (z for abundance and richness; t for Shannon diversity) and p-values derived from mixed-effects models (Generalised Linear Mixed Models for abundance and richness, and Linear Mixed Model for Shannon diversity). Values for abundance and richness are presented on the log scale. Significant seasonal differences are shown in bold.

10.7717/peerj.21622/supp-13 Supplemental Information 13 Macro-invertebrate sampling across bat-occupied and unoccupied caves in Mauritius Each row represents an individual macro-invertebrate record collected using pitfalls, quadrat (direct observation), and sticky traps at different distances from the cave entrance till deeper inside each cave.

## Additional Information and Declarations

### Field Study Permissions

The following information was supplied relating to field study approvals (*i.e.*, approving body and any reference numbers): Research and field permissions were approved by Forestry Services (FD No. 971/A/III) and National Parks and Conservation Services (NP 46/3V4) of the Ministry of Agro-Industry and Food Security.

### Data Availability

The following information was supplied regarding data availability: The data is available in the Supplemental Files.
