# Supplementary material for: An endangered cave-roosting insectivorous bat supports strongly distinct macro-invertebrate communities in caves of an oceanic island
Source: PeerJ. 2026 Aug 12;14:e21622. doi: 10.7717/peerj.21622 (PMC13477149; doi:10.7717/peerj.21622)
Supplement: Supplementary file 1 [file peerj-14-21622-s010.docx]

| **Locality** | **Elevation (m)^1^** | | **Annual rainfall (mm)^1^** | **Geological age (Ma)^2^** | **Entrance area (m^2^)** | **Cave length (position of roost within cave) (m)** | **Guano estimated size (m^2^)** |
| --- | --- | --- | --- | --- | --- | --- | --- |
| Gros Bois | | 208 | 2,720 | 0.7-0.03 | 46.2 | 60 (47, 55, 60) | 1.35, 1.75, 1.30 |
| Petit Raffray | | 39 | 1,333 | 0.7-0.03 | 13.6 | 80 (42) | 8.84 |
| Plaines des Roches (“Twilight Cavern”) | | 36 | 1,390 | 0.7-0.03 | 224.8 | 407 (60, 69, 80, 91, 132, 230, 263, 271, 280, 294) | 0.15, 0.33, 1.40, 1.28, 0.28, 2.21, 6.46, 8.05, 2.79, 3.85 |
| Quatre Bornes (Palma) | | 287 | 1,367 | 3.5-1.90 | 34.9 | 120 (37) | 13.02 |

^1^Intrapolated from Williaime, 1983; ^2^Following Baxter, (1976, 1975, 1972) and McDougall and Chamalaun, (1969)
