# Supplementary material for: An endangered cave-roosting insectivorous bat supports strongly distinct macro-invertebrate communities in caves of an oceanic island
Source: PeerJ. 2026 Aug 12;14:e21622. doi: 10.7717/peerj.21622 (PMC13477149; doi:10.7717/peerj.21622)
Supplement: Supplementary file 2 [file peerj-14-21622-s011.docx]

| **Diversity metric** | **Cave category** | |  |  |
| --- | --- | --- | --- | --- |
|  | With bat guano | Without bat guano | t value | p value |
| Abundance | 821.89 ± 227.41 | 196.73 ± 31.96 | 5.84 | 0.009 |
| Richness | 5.03 ± 0.14 | 2.36 ± 0.06 | 5.60 | 0.001 |
| Chao1 | 14.81 ± 0.82 | 12.14 ± 0.96 | 4.75 | 0.003 |
| Diversity (Shannon) | 1.01 ± 0.02 | 0.53 ± 0.02 | 4.21 | 0.009 |
