# Supplementary material for: An endangered cave-roosting insectivorous bat supports strongly distinct macro-invertebrate communities in caves of an oceanic island
Source: PeerJ. 2026 Aug 12;14:e21622. doi: 10.7717/peerj.21622 (PMC13477149; doi:10.7717/peerj.21622)
Supplement: Supplementary file 3 [file peerj-14-21622-s012.docx]

|  |  | **Season** | |  |  |
| --- | --- | --- | --- | --- | --- |
| **Metric** | **Cave category** | **Wet (Mean ± SE)** | **Dry (Mean ± SE)** | **Contrast (Wet - Dry)** | **p-value** |
| Abundance | With bats | 6.12 ± 0.33 | 6.23 ± 0.33 | -0.11 ± 0.08 | 0.168 |
|  | Without bats | 5.19 ± 0.33 | 5.10 ± 0.33 | 0.10 ± 0.08 | 0.255 |
| Richness | With bats | 2.64 ± 0.13 | 2.66 ± 0.13 | -0.02 ± 0.03 | 0.535 |
|  | Without bats | 2.48 ± 0.13 | 2.24 ± 0.13 | 0.23 ± 0.03 | **<0.001** |
| Shannon diversity | With bats | 1.83 ± 0.12 | 1.84 ± 0.12 | -0.01 ± 0.03 | 0.672 |
|  | Without bats | 1.63 ± 0.12 | 1.31 ± 0.12 | 0.32 ± 0.03 | **<0.001** |
